# HIV self-testing and the global diagnostic gap: Addressing a decade of missed opportunity

**DOI:** 10.1371/journal.pmed.1005241

**Published:** 2026-09-16

**Authors:** Reuben Granich, Somya Gupta, Isabelle Munyangaju, Mike Ruffner

**Affiliations:** 1 Independent Global Health Consultant, Geneva, Switzerland; 2 Independent Global Health Consultant, New Delhi, India; 3 Tinpswalo Research Association to Fight AIDS and TB, Chokwe, Mozambique; 4 Nucleo de Pesquisa Pediatria, Faculdade de Medicina, Universidade Eduardo Mondlane, Maputo, Mozambique; 5 Independent Global Health Consultant, Washington, DC, United States of America; McGill University Faculty of Medicine and Health Sciences, CANADA

## Abstract

- In 2015, a survey-based analysis across 16 sub-Saharan African countries estimated that nearly half of people living with HIV were undiagnosed, implying ~11 million people in the region were outside the treatment cascade. However, HIV self-testing (HIVST), a technology effectively positioned to close this gap, was deployed at negligible scale for a decade following WHO recommendation in 2016. The gap has narrowed, but through conventional testing rather than HIVST, and 2.6 million people in sub-Saharan Africa and 4.9 million globally still did not know their HIV status in 2025. The decade of delay carries a likely avoidable burden of millions of illnesses, deaths and onward transmissions, though this has not been formally modelled.
- The need for HIVST in low- and middle-income countries was estimated at 177 million self-tests in 2020, rising to 192 million by 2025. Confirmed procurement over the same period reached only 21 million kits in total, an average of 5.3 million per year, or 3% of estimated annual need.
- Four interlocking structural failures explain this missed opportunity: 1) research capture through demonstration projects that substituted for population-scale deployment; 2) the early supervised self-testing paradox in which provider-assisted delivery reconstituted the clinic barriers HIVST was designed to bypass; 3) procurement conservatism that suppressed market development; and 4) an accountability failure with no publicly accessible Africa-level volume series, making the scale of the missed opportunity impossible to measure from published data.
- Four actions could improve access: 1) mandatory public reporting of HIVST commodity volumes; 2) a dedicated Africa-specific distribution target of at least 50 million kits per year within the UNAIDS 95-95-95 framework; 3) community-based distribution as the default model for the general population; and 4) explicit HIVST volume commitments in the GHSD’s 2026–2030 memoranda of understanding and work plans.

## Abstract 2

In this Policy Forum article, Isabelle Munyangaju and colleagues discuss why the roll-out and scale-up of HIV self-testing over the last decade has been inadequate, and highlight what is needed—learning from past failures around governance, distribution and financing—to close the diagnostic gap.

## Introduction

Antiretroviral therapy (ART) suppresses HIV replication and prevents AIDS and onward transmission while preserving immune function and restoring near-normal life expectancy [1–3]. In 2000, the Rakai cohort in Uganda first suggested this by finding no linked transmissions below 1,500 copies of HIV per millilitre [4]. In 2006, Montaner and colleagues first called publicly for expanded ART access to reduce transmission and curb epidemic growth [5] and subsequent modelling showed that universal voluntary testing with immediate treatment could eliminate HIV in South Africa by driving incidence below one case per 1,000 per year [6]. In 2011, the HPTN 052 trial confirmed a 96% reduction in linked transmission with early ART initiation [7] and the PARTNER, PARTNER 2, and Opposites Attract studies subsequently documented zero genetically linked transmissions where suppression was maintained [8–10]. Undetectable equals untransmittable (U = U) built on those earlier calls for treatment as prevention [5], and by 2019 rested on one of the strongest bodies of evidence in infectious disease epidemiology [11].

**Fig 1:**
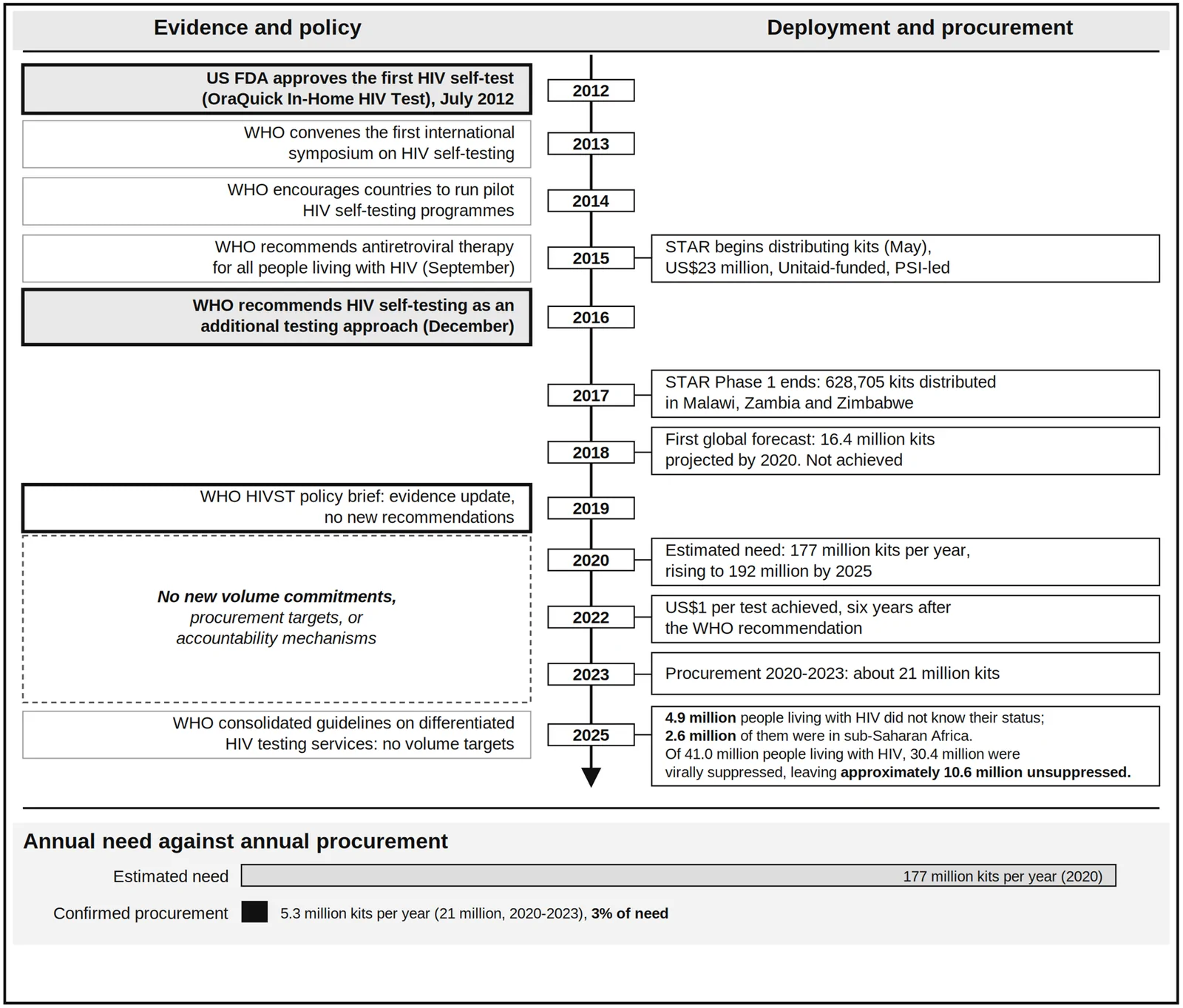
The missed opportunity to scale HIVST: evidence, policy and deployment, 2012–2025. A two-track timeline of the HIVST response. The left track shows evidence and policy milestones from US Food and Drug Administration approval of the first self-test in 2012 through successive World Health Organization (WHO) recommendations, including the period in which no volume commitments, procurement targets, or accountability mechanisms were set. The right track shows deployment and procurement over the same period. The lower panel compares estimated annual need with confirmed procurement, drawn to scale. The figure illustrates how substantial scientific and policy advances were not matched by population-scale implementation. The 2025 entry reports undiagnosed and viral suppression estimates for that year; ~10.6 million unsuppressed is derived by subtracting the 30.4 million with suppressed viral loads from the 41.0 million people living with HIV [13]. Compiled from US Food and Drug Administration approval records [14]; WHO HIVST guidance and policy documents [15–17]; Unitaid and WHO market and technology landscape reports [18,19]; STAR programme implementation data [20]; national programme data from eight African countries [21]; and UNAIDS epidemiological estimates [13].

Diagnosis is the gateway to treatment, survival and other prevention interventions. In 2014, the Joint United Nations Programme on HIV/AIDS (UNAIDS) introduced the 90-90-90 target: 90% of people living with HIV diagnosed, 90% of those diagnosed on ART and 90% of those on ART virally suppressed by 2020 [12]. The target linked diagnosis, treatment, and suppression for the first time and made closing the diagnostic gap the rate-limiting step in the prevention cascade [12]. In 2015, WHO issued consolidated guidelines on HIV testing services and recommended immediate ART for all people diagnosed with HIV regardless of CD4 count; WHO’s dedicated guidance endorsing HIV self-testing (HIVST) followed in December 2016. However, despite the millions of people undiagnosed, the technology best positioned to close that gap—HIVST—was deployed cautiously, constrained by research protocols, supervised delivery mandates and procurement timidity. Figure 1 summarises the sequence from the 2012 approval of the first self-test through successive WHO recommendations to the deployment that did not follow.

In 2015, when WHO recommended ART for all people living with HIV (PLHIV) regardless of CD4 count, pooled population-based survey analyses from 16 sub-Saharan African countries estimated that 46% of PLHIV remained undiagnosed [22]. Applied to the 24.1 million PLHIV in the region that year, this implies ~11 million people [13]. Population-based impact assessments confirmed that diagnosis translated reliably into treatment and suppression. In Zambia in 2016, 87% of diagnosed individuals were on ART and 89% of those virally suppressed [23], with similar results in Kenya in 2018 [24]. Diagnosis rather than linkage to care was the operative bottleneck and HIVST was a new technology designed to close it. HIVST provides an important option as an individual collects their own specimen of oral fluid or blood, performs a rapid diagnostic test and interprets the result themselves in private without clinic attendance or interaction with a healthcare worker [15].

The gap has since narrowed. However, by 2025 an estimated 2.6 million PLHIV in sub-Saharan Africa still did not know their status, while 1.5 million in eastern and southern Africa and 1.1 million in western and central Africa (22%) remain undiagnosed [13]. These gains took a decade and came through conventional facility-based testing at considerable cost. An inexpensive self-administered diagnostic was available throughout but was never deployed at a volume capable of accelerating it. The question this Policy Forum addresses is not whether the gap narrowed but how much faster it could have narrowed and at what cost in missed diagnoses, delayed treatment and preventable transmissions. Learning from these missed opportunities, we recommend policy actions to expand access and close the remaining gap.

## The promise of HIV self-testing

Conventional facility-based testing requires disclosure of risk behaviours to a provider, navigation of health systems and in many settings disclosure of serostatus to household members. Self-testing removes each of these barriers, since a person who cannot or will not test in a clinic may test at home, at a workplace or through a trusted peer. In July 2012, the US Food and Drug Administration approved the OraQuick In-Home HIV Test, the first over-the-counter HIV self-test [14]. In 2016, WHO endorsed self-testing as an additional approach, citing its potential to reach people reluctant to present to facilities and to enable testing through community networks, pharmacies, workplaces and peer distribution [15].

The prevention benefits of knowing one’s status predate universal treat-all policies. Among serodiscordant couples in Rwanda condom use rose from 4% to 57% within 12 months of disclosure, and HIV incidence among susceptible partners fell substantially without antiretroviral treatment [25]. A randomised trial across Kenya, Tanzania and Trinidad found greater reductions in unprotected intercourse after voluntary counselling and testing than after health information alone [26]. Delayed deployment therefore delayed diagnosis and treatment and forfeited behaviour change prevention.

WHO convened the first international symposium on self-testing in 2013 [15]. The principal implementation effort that followed was the Self-Testing AfRica (STAR) project, whose evidence directly informed the December 2016 guidelines that recommended HIVST [26]. Global volumes nonetheless remained far below need (see Box 1 and Table 1).

**Table 1:** Global procurement and deployment of HIVST in numbers.

Year	Confirmed procurement (million kits)	Estimated need (million tests)	% of need
2018	4.50	—	—
2019	5.03	—	—
2020	6.47	177.1	3.7%
2021	8.46	180.2	4.7%

**Box 1. Procurement and deployment of HIVST**

**Volumes against need**

Confirmed procurement reported to WHO and Unitaid reached only a small fraction of estimated need each year (Table 1). The July 2018 market forecast had projected at least 5 million kits by the end of that year and 16.4 million annually by the end of 2020; actual 2020 volume was 39% of that projection [18,27]. Commitments as of October 2020 totalled ~21 million kits for 2020–2023, an average of 5.3 million per year [18].

**How need was modelled**

WHO and Unitaid modelled HIVST need for 108 LMICs at 177.1 million tests in 2020, rising to 191.9 million by 2025 [18], covering populations WHO recommends for self-testing. The estimate measures recommended testing volume, not the number of people undiagnosed.

**National programme data**

Self-test kits distributed as a share of total testing volume ranged from 63% in Lesotho to under 1% in Kenya, with Zimbabwe, Malawi, Tanzania, and Uganda between 14% and 25% [21]. The share is sensitive to a shrinking denominator as much as to self-testing growth: in Lesotho, conventional testing fell 78% between 2019 and 2023 while total testing volume per capita fell by more than half over the same period [21].

**Volumes outside public programmes**

Commercial online sales in China alone reached 7.8 million kits in 2020, exceeding global confirmed public-sector procurement that year and confirming that demand and technology were not the constraint [28,29].

**Costs and financing**

Two costs are reported and frequently conflated: the manufacturer’s price, paid at the factory, and the delivered cost, which includes transport, staff time, and confirmatory testing. WHO and Unitaid costed the funding required to meet modelled need at US$1,321 million for 2020 [18]. At the US$2.00 manufacturer price then available, the commodity component of full sub-Saharan African need would have cost ~US$116 million, about 2% of the US$5.4 billion the US President’s Emergency Plan for AIDS Relief (PEPFAR) appropriated for bilateral HIV programmes in FY2024 [18,30]. The US$1 manufacturer price was not reached until July 2022, 6 years after WHO endorsement [31].

**The STAR initiative**

The US$23 million STAR project, funded by Unitaid and led by Population Services International, distributed 628,705 kits across Malawi, Zambia, and Zimbabwe in Phase 1 (2015–2017) [20].

## Four interlocking failures behind a decade of inadequate scale-up

The inadequate scale-up of HIVST between 2015 and 2023 reflected four mutually reinforcing structural failures.

### Research capture

WHO and the STAR initiative generated implementation evidence, informed WHO guidance and demonstrated the feasibility and acceptability of HIVST across multiple African countries. Despite supporting regulatory approval, national policy development, and market awareness among governments and donors in multiple countries, STAR’s efforts left global volumes at a fraction of estimated need. Its mandate was evidence generation through phased implementation rather than nationwide expansion, so it did not generate the financing, procurement, health system integration and national policy change that sustained large-scale implementation required. An evaluation commissioned by Unitaid found that global scale-up had not been achieved and market volumes had not diversified, with most self-tests priced too high for low- and middle-income countries (LMICs) [32]. COVID-19 further diverted resources and interrupted supply chains [18]. STAR’s stated objective was to accelerate global access and scale-up [27], and it delivered the evidence and the policy that scale-up required, but the kits did not follow.

### The early supervised self-testing paradox

In the early years after WHO endorsement, many implementation studies, demonstration projects and national programmes emphasised provider-assisted HIVST in which a healthcare worker demonstrated test use. Testing in private, without a provider, was what defined self-testing. By treating provider-assisted delivery as worth evaluating in its own right, researchers reduced self-testing’s original defining feature, privacy, to one variable among several delivery models to be compared. Assisted delivery accumulated evidence that unassisted community distribution at scale never generated, making supervision the better-evidenced option and unsupervised use the practice that had to be justified. The systematic review underpinning WHO’s 2016 recommendation set the framing by evaluating assisted and unassisted delivery as variants of a single intervention [33]. The resulting guidelines described privacy as a frequent rather than a defining feature and named directly assisted self-testing as a category [13], and donor monitoring made that category a reportable, if optional, disaggregate [34]. Cumulatively, these steps moved privacy from definition to optional attribute and made provider presence unremarkable. Twelve of the 13 national policies in Table 2 carry an assisted option and none identify unassisted delivery as its default. An evaluation of Unitaid’s portfolio identified highly medicalised services, with clinicians acting as gatekeepers of a new technology, as a barrier in West and Central Africa [54].

**Table 2:** HIV self-testing policy adoption and implementation characteristics in the 15 African countries with the highest HIV burden.

Country	PLHIV (2025)	Undiagnosed, *n* (%) (2025)	National instrument	Recommendation	Age and population	Distribution models	Assisted or unassisted	References
Angola	370,000	111,000 (30%)	No guidelines	—	—	—	—	—
Botswana	350,000	7,000 (<2%)	HIV clinical care guidelines 2023	Recommended	All, general population	Public facilities, private pharmacies	Assisted or unassisted; assisted by healthcare workers for children and adolescents	[35]
Cameroon	540,000	81,000 (15%)	HIVST framework 2020; HIV guidelines 2021	Recommended (phased implementation)	Age not specified; partners of people living with HIV or STIs, pregnant women, female sex workers, key populations, healthcare workers, young women, frequent retesters	Public facilities, community, secondary distribution	Assisted or unassisted	[36,37]
Côte d’Ivoire	NP	NP	National HIV strategic plan 2021–2025	Included in the strategic plan	Age not specified; key populations	Public facilities, community, secondary distribution, digital	Assisted (community-focussed, by peer educator or counsellor)	[38]
DRC	570,000	154,000 (27%)	HIV guidelines 2016	No HIVST recommendation	—	—	—	[39]
Ethiopia	660,000	86,000 (13%)	HIVST implementation manuals 2019 and 2020	Recommended (phased implementation)	Age not specified; key and priority populations	Public facilities, community, secondary distribution	Assisted, then assisted and unassisted	[40,41]
Kenya	1,500,000	105,000 (7%)	ART guidelines 2016; HIVST guidelines 2017	Recommended	All, general population	Facilities, pharmacies, community, vending machines, internet, secondary distribution	Assisted or unassisted	[42,43]
Malawi	1,000,000	40,000 (4%)	HIVST operational guidelines 2018	Recommended	—	—	—	[44]
Mozambique	2,500,000	250,000 (10%)	HIVST guideline (Guião) 2019	Recommended	≥15 years (age of assent); all, with priority to men, adolescents, key populations, partners of PLHIV	Public facilities, pharmacies, community and NGOs, secondary distribution, workplace	Assisted or unassisted	[45]
Nigeria	2,000,000	400,000 (20%)	HIVST operational guidelines 2019; national HIV guidelines 2020	Recommended	All, general population	Public facilities, pharmacies, community	Assisted or unassisted; assisted by healthcare workers for ages 2–11 years	[46,47]
South Africa	7,600,000	304,000 (4%)	National HTS policy 2016; HIVST guidance 2017	Recommended	All, general population	Health facilities, pharmacies, community, digital, vending machines, secondary distribution	Assisted or unassisted	[48]
Tanzania	1,800,000	144,000 (8%)	HIV and AIDS (Prevention and Control) Act (R.E. 2020); HTS guidelines 2021	Recommended	≥15 years; young and adult general population	Public facilities and registered sellers	Assisted or unassisted	[49,50]
Uganda	1,500,000	90,000 (6%)	HTS policy addendum 2018	Recommended	≥18 years; general population, with focus on young people, men, key populations	Public facilities, community, pharmacies, digital, vending machines, secondary distribution	Assisted or unassisted	[51]
Zambia	1,300,000	52,000 (4%)	Consolidated HIV guidelines 2018	Recommended	Age not specified; general population, with focus on key populations, men, adolescents, serodiscordant couples	Not specified	Assisted or unassisted	[52]
Zimbabwe	1,300,000	65,000 (5%)	ART guidelines 2016	Recommended	≥16 years; general population	Public facilities, community, pharmacies	Assisted or unassisted	[53]

Policy characteristics compiled from a review of national HIV policy and guidance documents; each national instrument is cited in the references column. PLHIV and undiagnosed estimates are UNAIDS epidemiological estimates, 2025 data [16]. PLHIV and undiagnosed values were verified against the UNAIDS 2026 estimates file; undiagnosed values are derived as PLHIV multiplied by the share not knowing their status. Abbreviations: NP, not published in the 2026 release. PLHIV, people living with HIV. HIVST, HIV self-testing. ART, antiretroviral therapy. HTS, HIV testing services. STI, sexually transmitted infection. NGO, nongovernmental organisation. DRC, Democratic Republic of Congo. R.E., revised edition.

Elements of this early caution persist. Angola and the Democratic Republic of Congo have no regulatory framework for self-testing, leaving ~265,000 undiagnosed people living with HIV without a legal route to a self-test (Table 2). Four of the 13 policies set an explicit age threshold: Tanzania (at 15 years) and Zimbabwe (at 16 years) match their national ages of consent for HIV testing, while Mozambique (at 15 years) and Uganda (at 18 years) exceed theirs, both 12 years, which excludes adolescents entitled to consent to a conventional test in two countries with 4.0 million PLHIV [21,28]. By 2018, only 4.1% of Kenyan adults who had ever tested had used a self-test [55], and across 21 sub-Saharan African countries, knowledge and use among 270,241 women was 2.17% in 2022 [56]. The paradox was institutionalised less through explicit restriction than through a category that made supervision available, respectable, and countable.

HIVST delivery has since evolved to include community-based, pharmacy-based, secondary and digital-supported models. Deployment remains stunted, and the critique here concerns the early period, when supervised delivery predominated in demonstration projects and shaped subsequent programme design [57–61].

### Procurement conservatism

The US PEPFAR [62], the Global Fund [63], and domestic health ministries treated HIVST as a niche adjunct to facility-based testing rather than a first-line population intervention. Between them, the Global Fund, PEPFAR and Unitaid accounted for 86% of confirmed self-testing volumes in 2020, so their purchasing decisions set the market [18]. The instruments to shape this market existed and were used elsewhere. Within months of the 2024 prevention results for the pre-exposure prophylaxis (PrEP) medication lenacapavir, PEPFAR and the Global Fund committed to supply up to 2 million people over 3 years, an arrangement the US Department of State described as market shaping [64]. No comparable commitment was made by the major purchasers for self-testing, and the volume guarantee that eventually lowered the price came from CHAI and MedAccess in 2022, 6 years after WHO recommendation. Low procurement volumes signalled weak demand to manufacturers, sustaining high unit prices, which in turn justified further low procurement.

### Balancing caution and missed opportunity

Cautious deployment was driven by early concerns about social harm, weak linkage to care, limited data capture for surveillance and the regulatory challenges of decentralised diagnostics [15,19,65]. Some were initially justified, but a decade of evidence shows that serious social harms are rare [20,27] and that linkage support, where offered, can be delivered through community-based, peer-led and digital models [15,33,54,57,58,66]. Digital tools and assured linkage are enhancements, not prerequisites for distribution. The home pregnancy test is an instructive illustration: it is sold through pharmacies and retail channels with no accompanying linkage infrastructure. A positive result is routinely confirmed when the person presents for clinical care, and pregnancy itself can carry heavy stigma. Yet no one proposes withholding pregnancy tests until a linkage mechanism is demonstrated.

Neither test is a diagnosis on its own. For HIV, confirmation within the national testing algorithm establishes the diagnosis before lifelong treatment begins, because misclassification means a regimen started in error [17]. These are reasons to integrate HIVST into national testing algorithms and to offer linkage support to every person who tests, not reasons to withhold kits. The obligation to establish and offer linkage pathways rests with the health system, not with the person testing. Requiring programmes to demonstrate a linkage mechanism before kits are distributed inverts the sequence. The risks of imperfect implementation are outweighed by the consequences of nondeployment in settings with large undiagnosed populations [12,22,65].

Caution was understandable in 2012 when the evidence base was thinner. A decade later, HIVST has been held to an unusually high standard of proof compared with the same test administered by a lay provider, while the harms of delayed diagnosis and the missed prevention opportunity went unmeasured [19,34,65]. Slow adoption was evident well before COVID-19, which amplified an implementation gap that already existed.

## Power, decision-making, and the geography of implementation

The trajectory of HIVST deployment also reflects where decision-making authority sits within the global HIV response. Funding priorities, procurement strategies and evidentiary thresholds have largely been set by bilateral donors, technical agencies, and multilateral financing mechanisms rather than by the governments and communities most affected. A technology designed to expand autonomy and access was therefore introduced through highly controlled delivery models, mediated by the same institutional structures that had historically constrained access to testing [15,20,27].

The cost of that ordering is now visible. The annual number of people newly learning their HIV status fell in each year from 2022, from 1.1 million to 845,000 in 2025, and the annual increase in people virally suppressed fell from 1.5 million in 2023 to 1.0 million in 2024 [13]. Diagnosis slowed first, and both trends preceded the decline in donor funding.

Rebalancing requires financing, but through re-prioritisation rather than new money. Closing the diagnostic gap has been a lesser priority within HIV budgets and addressing it will take push from donors and pull from countries. The push is under way. From its 2024 funding cycle, the Global Fund required all country HIV funding requests to include and report on self-testing, and its procurement rose from 0.22 million kits in 2018 to 5.25 million in 2021, alongside 4 million procured by PEPFAR [32]. The pull is authority at country and community level to define distribution models, set procurement priorities and volume targets, align age thresholds with national consent ages (Table 2), and open over-the-counter and private pharmacy distribution with or without subsidised pricing, so that testing is normalised rather than mediated through the clinic. Earlier diagnosis averts the costlier downstream burden of illness, death and onward transmission, and increasing the number of people virally suppressed sooner, rather than waiting for individuals to present at a clinic, is central to epidemic control [12]. A passive, facility-based approach alone is unlikely to close the last mile gap of undiagnosed PLHIV.

## The surveillance failure

The programmatic failures were compounded by an accountability failure of comparable magnitude. Neither WHO 2016 recommendation, the 2019 evidence update, nor the 2024 consolidated guidelines set a distribution volume target or established any accountability mechanism for shortfalls [15–17]. Nor do any of the 15 national policies (Table 2). Median time from the December 2016 WHO recommendation to national policy adoption was 3 years, ranging from within the same year in Kenya and Zimbabwe to 7 years in Botswana. Two of the 15 countries, Angola and the Democratic Republic of Congo, had adopted no HIVST policy as of August 2026 (Table 2). Policies permitting unassisted distribution for the general population cover ~10.5 million of the ~23 million PLHIV in these countries (Table 2). The technology, evidence and normative guidance were all in place from 2016; what varied was the speed at which countries converted guidance into policy. Neither WHO nor the major funders attached any timeline, target or reporting requirement to that conversion.

No routine surveillance system for self-testing volumes was ever built. The first compilation of national programme data appeared in 2026, a decade after the recommendation, and it was a research exercise covering only government-distributed kits in eight countries [21]. PEPFAR introduced the HTS_SELF indicator, yet aggregate Africa-level totals have never appeared in any publicly accessible report [67]. WHO, UNAIDS and the Global Fund track testing coverage outcomes rather than commodity volumes. How many self-tests were distributed in sub-Saharan Africa in any given year could not be answered from published data for a decade and can now be answered only partially [15,21,65]. An intervention that is not counted is not managed.

Without denominator data on self-tests distributed it is not possible to assess coverage relative to need, identify inequities in access or evaluate the efficiency of distribution models. PrEP, recommended by WHO one year before self-testing, has been tracked since 2016 through a public global tracker reporting initiations quarterly by country and product [68]. Self-testing has no equivalent, a disparity that reflects a hierarchy of programmatic attention in which diagnosis remains under-monitored despite being the gateway to every subsequent outcome including viral suppression.

## Quantifying the cost of delay

The consequences of limited HIVST deployment are structurally predictable (see Box 2 and Table 3). Each missed diagnosis is a cascade that never begins, with no treatment, no preservation of immune function and no reduction in transmission risk. Across a decade of inadequate deployment in a population where ~11 million PLHIV in sub-Saharan Africa were undiagnosed in 2015 [13,22], the likely avoidable burden runs to millions of illnesses, deaths and onward transmissions [22]. This follows from the size of the undiagnosed population and the established relationship between diagnosis, treatment and prevention [5–7,22], but it has not been formally modelled. Formal modelling of this counterfactual is needed to inform planning under the 2026–2030 memoranda of understanding through which the US Bureau of Global Health Security and Diplomacy (GHSD), which administers PEPFAR, is transitioning bilateral HIV programmes to country ownership under the America First Global Health Strategy [7,71].

**Table 3:** Costs of HIVST kits.

Year	Price (US$ per kit)	Basis
2020	1.99–6.00	Ex-works range across products [18]
2020	~2.00	Two most widely available products [18]
2022 (July)	1.00	Volume guarantee, CHAI/MedAccess [58]
2026	from 0.90	Quality-assured products, ex-works [69]
2026	0.90–3.59	Global Fund pooled procurement range [70]

As of 2025 ~4.9 million PLHIV globally remained undiagnosed, the majority in sub-Saharan Africa [13]. This gap has persisted for more than two decades into the treatment era and after more than US$94 billion budgeted for the HIV response in 40 sub-Saharan African countries between 2002 and 2021 [72]. The gap remains nearly a decade after WHO endorsed a technology well suited to close it. That is institutional failure and not scientific uncertainty or resource constraint.

Resources are now genuinely constrained. Donor government funding for HIV in LMICs fell to US$6.2 billion in 2025 from US$8.3 billion in 2024, a 25% decline and the lowest level since 2007 [73]. Constraints make the choice of intervention more consequential. Closing the diagnosis and suppression gap is the highest priority. Of 41.0 million PLHIV, around 10.6 million are not virally suppressed, of whom 4.9 million have never been diagnosed [13]. Diagnosis is the entry point for that group and self-testing may be the least expensive way to reach it at US$0.90 to US$3.59 per kit under current pooled procurement (Box 2 and Table 3). The 2026–2030 GHSD memoranda currently being negotiated will set the architecture within which choices around the prioritisation of HIVST will be made.

**Box 2. Finance mechanisms and throughput for HIVST**

Most HIV testing is embedded within facility-based service delivery, donor-supported programmes and health system financing mechanisms. Self-testing was not only a new diagnostic technology. It moved the work of testing out of the facility and onto the individual, who performed it unpaid, a form of outsourcing that threatened the patient contact, reimbursement and throughput on which facilities and implementing partners were assessed. Self-testing was consistently framed as an additional approach that complements existing services. That framing was accurate, and it may also have been convenient. An intervention defined as supplementary displaces no budget line, acquires no constituency to defend it, and may therefore have remained an orphan intervention funded at the margin. The efficiency case, however, rested on displacement.

Self-testing carries a behavioural prevention benefit from knowledge of status alone, a clinical benefit from earlier treatment initiation, and a transmission benefit from earlier viral suppression [6–9]. It also removes nonreactive testers from the facility queue, concentrating provider time on confirmatory testing and linkage [27]. Both mechanisms substitute for facility throughput, which is what funding, targets and reporting rewarded. Aligning financing, indicators and procurement to reward diagnosis regardless of where testing occurs could remove this source of institutional resistance. A system that will not purchase a technology which performs its labour for free is not economising. It is defending control over the diagnosis.

An evaluation commissioned by Unitaid identified the mechanism sustaining low procurement. Donors and at least one country stakeholder regarded the self-test’s higher price relative to a conventional rapid diagnostic test, ~US$0.70, as a bottleneck to market growth where testing budgets are finite [33]. Where a budget is fixed, substituting a self-test at roughly three times the unit price reduces the number of tests it can buy, so a manager accountable for testing volume rationally keeps self-testing marginal absent an expanded budget or a mandate to reallocate. That price gap is trivial against the cost of a missed diagnosis and against overall HIV budgets. The full commodity cost of meeting estimated sub-Saharan African need is about 2% of PEPFAR’s FY2024 bilateral HIV appropriation (Box 1). Research-scale procurement priced a self-test at US$3.50 to US$16 per test in 2015 [74], falling to US$2 to US$12 in the public sector by 2018 [19] and then stalling near US$2 through 2020 [18]. The US$1 price achieved in July 2022 through a volume guarantee [58] shows that further reductions were available years earlier (Table 3).

The unit economics reinforce the pattern. WHO and Unitaid’s costed estimates reflect pilot-scale programmes distributing only 6.5 million kits globally in 2020, when fixed costs such as demand creation and training would be expected to fall with volume. Retail and pharmacy distribution bypasses programme funding entirely. Online sales of 7.8 million kits through two Chinese platforms in 2020 exceeded the total volume procured by all donors worldwide that year [18,28]. No published estimate of delivered cost at population scale exists, because that scale was never reached.

## Actions to increase access and reduce the HIV diagnosis gap

### Mandatory reporting of distribution volumes

WHO could establish a deliberately simple HIVST indicator framework built on one core measure, self-test kits distributed per year by country, modelled on PEPFAR’s HTS_SELF indicator [67] and reported through routine systems such as DHIS2 rather than parallel surveys [32]. GHSD can embed this within the co-financing benchmarks of the 2026–2030 memoranda, and the Global Fund can make it a condition of grant performance reporting [32]. National returns would aggregate to published annual totals, giving self-testing the equivalent of the public tracker PrEP has had since 2016 [68]. One number answers the accountability question.

### A dedicated Africa-specific distribution target

WHO and UNAIDS could establish an HIVST sub-target within the 95-95-95 framework, for example a minimum of 50 million kits per year across sub-Saharan Africa, roughly three quarters of the 67.5 million tests modelled as regional need for 2025 (Box 1). The target should count kits reaching users through private pharmacy, retail, and online channels as well as public sector volumes. Where retail prices exceed what users will pay, subsidies or price guarantees can bring kits within reach while leaving distribution to commercial infrastructure [32]. Shortfalls are multifactorial, so the appropriate consequences are procedural rather than punitive. Each institution’s share would be named in advance. WHO would be responsible for normative tracking, the Global Fund and PEPFAR for procurement financing, and national programmes for distribution and for the regulatory changes that permit retail sale. Shortfalls would be reported publicly every year within existing grant and programme performance reviews, alongside a published corrective plan. Such a mechanism assigns ownership of the gap without assigning blame for it.

### Abolition of supervised self-testing as the default model

Community-based distribution, pharmacy dispensing, workplace programmes, secondary peer-to-peer distribution and vending access must become the default for the general population. Countries should incorporate HIVST into national HIV testing algorithms so that reactive results trigger standardised confirmatory testing and linkage to care regardless of where testing occurs. Financing and performance indicators should jointly incentivise facilities, community programmes and pharmacies to maximise diagnosis and linkage rather than compete for testing volumes.

### Leveraging current financing and policy opportunities

**Fig 2:**
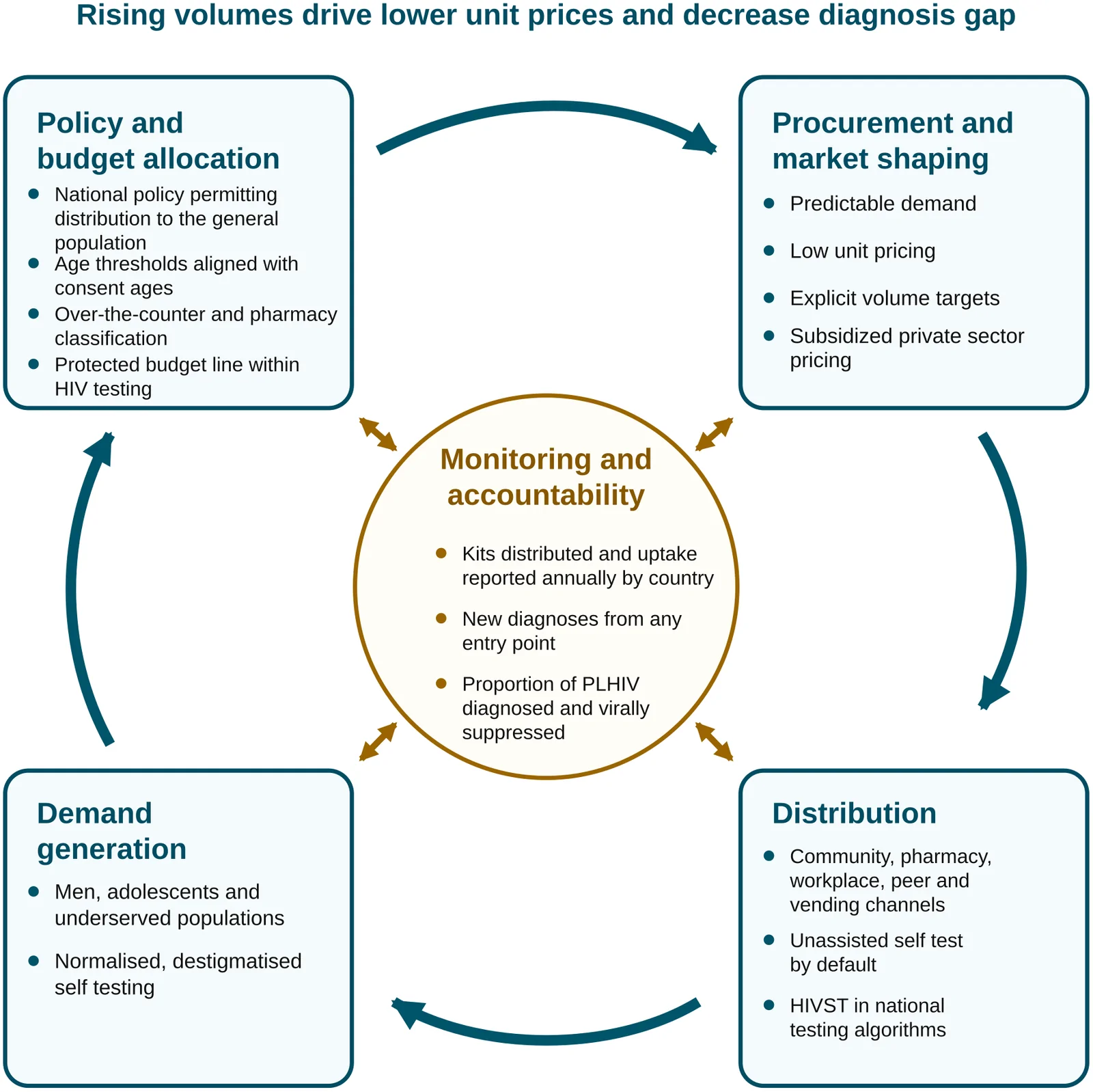
Operational pathway for population-scale HIV self-testing. Four domains cycle around a central monitoring and accountability function. Policy and budget allocation determine whether self-tests may be sold, to whom and through which channels, and whether a protected budget line exists within HIV testing. Procurement and market shaping establish predictable demand, low unit pricing and explicit volume targets. Distribution moves kits through community, pharmacy, workplace, peer and vending channels, unassisted by default for the general population and integrated into national testing algorithms. Demand generation reaches men, adolescents and underserved populations and normalises testing. The cycle closes because rising volumes lower unit prices and normalised demand strengthens the case for permissive policy. Monitoring and accountability exchange information with every domain: kits distributed and uptake reported annually by country, new diagnoses from any entry point, and the proportion of people living with HIV diagnosed and virally suppressed. Because reactive self-tests require confirmatory testing at services, new diagnoses provide a common endpoint for self-testing and routine testing alike.

The ongoing GHSD 2026–2030 memoranda negotiations are one opportunity to incorporate explicit HIVST procurement and distribution commitments. More broadly, HIVST scale-up should be embedded within national HIV strategic plans, Global Fund grant cycles, domestic financing mechanisms and other investment frameworks. Explicit procurement targets, predictable funding and accountability for implementation will be essential to population-scale access. Figure 2 summarises an operational pathway for translating these recommendations into implementation.

## Conclusions

The delayed rollout of HIVST is not a failure in the abstract. Knowing one’s HIV status is the first step to treatment and to protecting others. When simple, effective, and affordable diagnostic tools exist but are not deployed at scale, the resulting millions of illnesses, deaths, and transmissions are preventable. The persistence of large undiagnosed populations is a strategic and technical failure, and a question of distributive justice and accountability. A simple, effective and privacy-preserving tool was constrained for nearly a decade by research capture, delivery models that undermined its purpose, procurement conservatism and an absence of accountability for commodity deployment.

Unanswered questions remain. What did the delay cost in infections, illnesses and deaths, and what does each further year of inadequate deployment cost? Which delivery models reach undiagnosed people most effectively and most equitably, and at what cost from the individual, health system and societal perspectives? Can digitally enabled reporting and navigation strengthen monitoring without eroding the privacy that makes self-testing attractive? None of these questions needs an answer before distribution expands.

Diagnosis is also where prevention begins. For those who test positive, treatment prevents onward transmission [5–9]. For those who test negative, self-testing supports condom use and, for people at substantial ongoing risk, the initiation and continuation of oral PrEP [17]. The current cycle of national strategy development, bilateral agreements and multilateral financing decisions is an opportunity to restore diagnosis as a central pillar of the response. That requires explicit volume targets, predictable procurement, community-based distribution as the default, and routine public reporting of testing commodities alongside outcomes. The science is established, the tools are available and the costs are modest. What remains is for WHO to set a target, for the Global Fund and PEPFAR to buy at scale, and for ministries to let people test without asking permission.

## Abbreviations

ART: Antiretroviral therapy
GHSD: Global Health Security and Diplomacy
HIVST: HIV self-testing
LMICs: low- and middle-income countries
PEPFAR: President’s Emergency Plan for AIDS Relief
PLHIV: people living with HIV
PrEP: pre-exposure prophylaxis
STAR: Self-Testing AfRica
UNAIDS: United Nations Programme on HIV/AIDS
WHO: World Health Organization.
